# Multiple Hürthle cell adenomas in a patient with thyroid hormone resistance

**DOI:** 10.1530/EDM-13-0032

**Published:** 2013-12-01

**Authors:** Gemma Xifra, Silvia Mauri, Jordi Gironès, José Ignacio Rodríguez Hermosa, Josep Oriola, Wifredo Ricart, José Manuel Fernández-Real

**Affiliations:** 1 Department of Diabetes, Endocrinology and NutritionHospital Dr Josep TruetaGironaSpain; 2 Department of SurgeryHospital Dr Josep TruetaGironaSpain; 3 Biochemistry and Molecular Genetics DepartmentCDB Hospital ClinicBarcelonaSpain; 4 CIBERobn, patophysiology of obesity and nutritionSpain

## Abstract

Background: Thyroid hormone resistance (RTH) is a rare cause of thyroid dysfunction. High TSH levels, as described in RTH syndrome, are known to be associated with an increased risk of developing thyroid nodules with subsequent growth and malignancy.

Patient findings: In 2006, a 29-year-old Caucasian man presented with a palpable mass in the neck. Increased free thyroxine and triiodothyronine levels were found in the context of unsuppressed TSH levels, despite no signs or symptoms of hyperthyroidism. Ultrasonography revealed a multinodular and enlarged goitre, and fine-needle aspiration cytology revealed suspicious features of malignancy. After excluding pituitary tumour and levothyroxine (l-T_4_) treatment, the patient was diagnosed with generalized RTH. Screening for all the known mutations in thyroid hormone receptor-β (*TR* *β* (*THRB*)) was negative. Thyroidectomy disclosed five Hürthle adenomas and three hyperplasic nodules. Euthyroidism was achieved after surgery with 6.1 μg/kg per day of l-T_4_.

Conclusion: RTH may be a risk factor that predisposes to the development of multiple Hürthle cell adenomas. To our knowledge, this is the first case of multiple Hürthle cell adenomas in a patient with RTH.

**Learning points:**

- High TSH levels, as described in RTH syndrome, are known to be associated with an increased risk of developing thyroid nodules, with subsequent growth and malignancy.
- The exact role of *TR* *β* mutants in thyroid carcinogenesis is still undefined.
- We report the first case of multiple Hürthle cell adenomas associated with RTH.

## Background

Thyroid hormone resistance (RTH) is an inherited syndrome, mostly inherited as an autosomal dominant trait, caused by mutations in the thyroid hormone receptor-β (*TRβ* (*THRB*)) gene and characterized by reduced sensitivity to thyroid hormone (TH). The precise incidence is unknown. In a limited neonatal screening for free thyroxine (fT_4_) in 80 000 newborn infants, RTH was detected in 1 of 40 000 live births (1).

Elevated serum TH levels in the setting of non-suppressed thyrotrophin (TSH) levels reflect the reduced response of target tissues. Two different forms of RTH have been described, the generalized and pituitary forms. Generalized RTH is the most prevalent form, in which patients are clinically euthyroid. Some subjects can exhibit signs of hyperthyroidism in one tissue and findings suggestive of hypothyroidism in other tissues according to the distribution of the different thyroid receptor subtypes. Goitre is reported to be the most common feature followed by sinus tachycardia. Other features are growth delay or attention deficit. Signs and symptoms vary in patients with the same mutation, even in the same family (2).

It is well known that high TSH levels, as described in RTH syndrome, are associated with an increased risk of developing thyroid nodules, with subsequent growth and malignancy (3), and with advanced stages of thyroid cancer (4). TSH has been reported to act as a growth factor in thyroid tissue. Despite the exact role of RTH in thyroid carcinogenesis being still undefined, three recent reports (5) (6) (7) have documented two cases of RTH associated with papillary thyroid carcinoma (PTC). To the best of our knowledge, we report the first case of multiple Hürthle cell adenomas associated with RTH.

## Case presentation

In July 2006, a 29-year-old Caucasian man without a family history of thyroid disease presented with a palpable and painless mass in the neck. The patient had a history of mild mitral valve insufficiency diagnosed at the age of 15 years and hypertension since 28 years, treated with atenolol 50 mg a day. Physical examination revealed blood pressure of 135/74 mmHg and heart rate of 80 b.p.m. His BMI was 23.6 kg/m^2^. Cervical examination revealed two palpable hard nodules with a mildly enlarged thyroid gland. No other abnormalities were found.

## Investigation

Increased fT_4_ and free triiodothyronine (fT_3_) levels were found in the context of unsuppressed TSH levels (Table 1). The determination of fT_4_ and fT_3_ levels was performed by electrochemiluminescent immunoassay using the equipment provided by Roche (Elecsys E170). Tests carried out for anti-thyroid receptor, anti-thyroglobulin and anti-thyroid peroxidase antibodies were negative. No alterations in lipid profile were found. Sex hormone-binding globulin and albumin levels were within the normal range.

**Table 1:** Changing levels of thyroid hormones and TSH pre and post surgery and before and after replacement therapy with levothyroxine

	**Baseline**	**After hemithyroidectomy** (without l-T_4_ treatment)	**After hemithyroidectomy** (with l-T_4_ 150)	**After total thyroidectomy** (l-T_4_ 300)	**After thyroidectomy** (l-T_4_ 450)
TSH (0.27–4.2 mIU/l)	2.12	5.44	3.88	30	2.2
FT_4_ (0.7–1.8 pmol/l)	3.06	3.08	3.68	3.39	4.7
FT_3_ (1.8–4.6 ng/dl)	8.35	7.42			5.55

FT_4_, free thyroxine; FT_3_, free triiodothyronine; TSH, thyrotrophin.

All first-degree relatives of the patient were studied. Tests carried out for thyroid antibodies were negative in all the cases. None of the relatives studied exhibited any alteration in thyroid function.

Familial dysalbuminaemic hyperthyroxinaema was unlikely due to an increase in not only total T_4_ levels, but also fT_3_ and fT_4_ levels, and especially due to the high levothyroxine (l-T_4_) doses required to maintain TSH levels within the normal range. TSH-secreting pituitary adenoma was ruled out, because the patient had no clinical features of thyrotoxicosis, a negative magnetic resonance imaging scan of the pituitary gland, and a normal level of the α-subunit of TSH (<0.25 mUI/ml, *n*: 0–0.8).

Thyroid ultrasonography revealed an enlarged thyroid gland with multiple well-defined nodules in the left lobe. The two biggest nodules measured 17×27 and 21×24 mm respectively. Fine-needle aspiration cytology (FNAC) of both the nodules revealed few follicular cells with microcalcifications. A left hemithyroidectomy was scheduled, and the pathological examination disclosed four Hürthle adenomas.

Two years later, thyroid ultrasonography revealed four new nodules in the right lobe. FNAC of the biggest one revealed follicular cells with oncocytic metaplasia. Removal of the remaining thyroid lobe disclosed Hürthle adenoma (8 mm) and three hyperplasic nodules.

Genomic DNA was isolated from the peripheral blood leukocytes of the patient. PCR amplification was conducted from axon 7 to axon 10 of the *TRβ* gene. The sequences were analysed using the Sequence Pilot Software (JSI Medical Systems, Kippenheim, Germany). Screening of all the known mutations in the *TRβ* gene was negative.

## Treatment

After surgery, the patient needed a dose of 450 μg/day (6.1 μg/kg per day) to maintain TSH levels comparable to the preoperative values and to be clinically euthyroid. Malabsorption was ruled out. His heart rate was 70–80/min.

## Outcome and follow-up

The patient is currently well.

## Discussion

Excluding other aetiologies, the patient was diagnosed with RTH. No other family member exhibited any thyroid dysfunction. *De novo* RTH-β mutations have been reported to be responsible for 27% of RTH cases (7). Genetic mutation has not been found in the current case. The genetic study includes the complete sequence of exons 7, 8, 9 and 10. Earlier, it has been reported that 15% of RTH cases have no *TRβ* mutations (7). The phenotype of these patients does not differ from that of cases with *TRβ* gene mutations, suggesting the involvement of other as-yet unidentified genes encoding the messengers along the intracellular pathways downstream to the receptor.

Of note, RTH in this patient was concomitant with the development of five Hürthle cell adenomas, a rare thyroid neoplasm of follicular cell origin, and hyperplasic nodules. Surgery is only rarely required in RTH. Total thyroidectomy was the best option as cytology was suggestive of malignancy.

Hürthle cell adenomas, also known as oncocytomas or oxyphilic thyroid adenomas, represent a rare subgroup of follicular thyroid lesions, surrounded by a thin capsule and characterized by cells with distinctive eosinophilic cytoplasm.

Three recent reports have documented three cases of RTH associated with PTC (5) (6) (7). The first case is a 38-year-old Korean woman with generalized RTH and incidental PTC (5). She presented with goitre and no signs or symptoms of hyperthyroidism. A missense mutation in exon 9 of the *TRβ* gene that causes the substitution of threonine with methionine at codon 310 was found. No family members were affected. FNAC revealed suspicious features of malignancy. After thyroidectomy, pathological examination revealed two micro-PTCs in both the lobes. She had no signs of hypothyroidism under treatment with 250 μg/day of l-T_4_ (5.1 μg/kg per day). The second case is a 29-year-old Brazilian man with a family history of RTH (6). He was diagnosed with RTH at the age of 15 years and his medical history included atrial fibrillation. Analysis of the *TRβ* gene revealed a missense mutation in exon 9 causing a change of the amino acid alanine to threonine at codon 317. Thyroidectomy was indicated after two unsatisfactory FNAC procedures. Intraoperative frozen-section analysis revealed PTC, and bilateral cervical lymph node dissection was performed. Pathological examination indicated multicentric papillary carcinoma without lymph metastasis. Signs of hypothyroidism improved under treatment with 400 μg/day of l-T_4_ (5.8 μg/kg per day). The third reported case is a 29-year-old woman with generalized RTH and incidental PTC (7). She presented with diffuse goitre and 5-year symptoms of palpitation and nervousness. Failure to respond to anti-thyroid drugs led to subtotal thyroidectomy. Pathological examination revealed an 8-mm PTC and total thyroidectomy was indicated. Screening of family members revealed two siblings with elevated serum fT_3_ and fT_4_ levels with normal serum TSH concentrations but no thyrotoxic manifestations. Sequencing of the *TRβ* gene revealed a single-nucleotide substitution resulting in the replacement of the normal methionine with a threonine at codon 334. She was clinically asymptomatic under treatment with 300 μg/day of l-T_4_.

It is important to consider mutations in the TSH receptor (*TSHR*) during differential diagnosis. A 33-year-old man presented with multinodular goitre (7). Thyroid ultrasound demonstrated a hypoechoic nodule measuring 8×10×8 mm with FNAC being compatible with PTC. Histopathological examination after total thyroidectomy with central lymph node dissection revealed a PTC focus of 12 mm in the longest diameter with capsular invasion. A novel nucleotide substitution resulting in the replacement of the normal isoleucine with phenylalanine (I364F) in the extracellular domain of the *TSHR* was described. *TSHR* mutation was incidental and did not correlate with the increased serum TSH levels or any of the thyroid test abnormalities in the subject. The required dosage of T_4_ was 250 μg/day. Seven family members were studied. Four exhibited the same nucleotide substitution with different phenotypes.

The exact role of RTH in carcinogenesis is still unknown. According to previous studies, TSH acts as a growth factor due to the high levels and an increased bioactivity of TSH as described in RTH (8). In the reported case, with respect to the normal values of TSH, it is possible that TSH acts as a growth factor through its increased bioactivity. In animal models, spontaneous development of thyroid follicular carcinoma has been described in mice homozygous for a target mutation in the *Trβ* gene (9). The metastatic thyroid cancer exhibited both anaplastic and follicular patterns (10). Molecular analyses in these mice revealed the activation of the cyclin 1–cyclin-dependent kinase-4–transcription factor E2F1 pathway, known to be associated with thyroid tumour cell proliferation (9). In addition, mutation of a single allele of the *TRβ* gene is also sufficient for follicular thyroid carcinoma to develop in mice treated with propylthiouracil, in which TSH levels increase even more (9). Recently, Franco *et al*. (11) have found that the TSH signalling pathway may predispose thyroid cells to BRAF-induced transformation in mice with a thyroid-specific knocking of oncogenic *Braf* (LSL-Braf^V600E^/TPO-Cre). However, thyroid cancer is still an uncommon occurrence in patients with genetically confirmed RTH, and it must be investigated.

There are some study limitations of the reported case. TRH test was not performed in this patient. None of the investigations reported have 100% sensitivity or specificity to exclude TSHoma either alone or in combination. Despite this limitation, patient outcomes support the diagnosis of RHT. Familial dysalbuminaemic hyperthyroxinaema was unlikely, given an increase not only in total T_4_ levels, but also in fT_4_ and fT_3_ levels, in the same proportion.

## Patient consent

The authors confirm that written informed consent was obtained from the patient.

## Author contribution statement

S Mauri is the named physician of the patient. G Xifra, S Mauri, J M Fernández-Real and W Ricart are co-authors of the article.
